# Genome-Wide Sex and Gender Differences in Cancer

**DOI:** 10.3389/fonc.2020.597788

**Published:** 2020-11-23

**Authors:** Camila M. Lopes-Ramos, John Quackenbush, Dawn L. DeMeo

**Affiliations:** ^1^ Department of Biostatistics, Harvard T.H. Chan School of Public Health, Boston, MA, United States; ^2^ Department of Data Science, Dana-Farber Cancer Institute, Boston, MA, United States; ^3^ Channing Division of Network Medicine, Department of Medicine, Brigham and Women's Hospital and Harvard Medical School, Boston, MA, United States; ^4^ Division of Pulmonary and Critical Care Medicine, Brigham and Women's Hospital, Boston, MA, United States

**Keywords:** sex, gender, cancer, genomics, genetics, epigenetics, gene networks

## Abstract

Despite their known importance in clinical medicine, differences based on sex and gender are among the least studied factors affecting cancer susceptibility, progression, survival, and therapeutic response. In particular, the molecular mechanisms driving sex differences are poorly understood and so most approaches to precision medicine use mutational or other genomic data to assign therapy without considering how the sex of the individual might influence therapeutic efficacy. The mandate by the National Institutes of Health that research studies include sex as a biological variable has begun to expand our understanding on its importance. Sex differences in cancer may arise due to a combination of environmental, genetic, and epigenetic factors, as well as differences in gene regulation, and expression. Extensive sex differences occur genome-wide, and ultimately influence cancer biology and outcomes. In this review, we summarize the current state of knowledge about sex-specific genetic and genome-wide influences in cancer, describe how differences in response to environmental exposures and genetic and epigenetic alterations alter the trajectory of the disease, and provide insights into the importance of integrative analyses in understanding the interplay of sex and genomics in cancer. In particular, we will explore some of the emerging analytical approaches, such as the use of network methods, that are providing a deeper understanding of the drivers of differences based on sex and gender. Better understanding these complex factors and their interactions will improve cancer prevention, treatment, and outcomes for all individuals.

## Introduction

**Figure 1:**
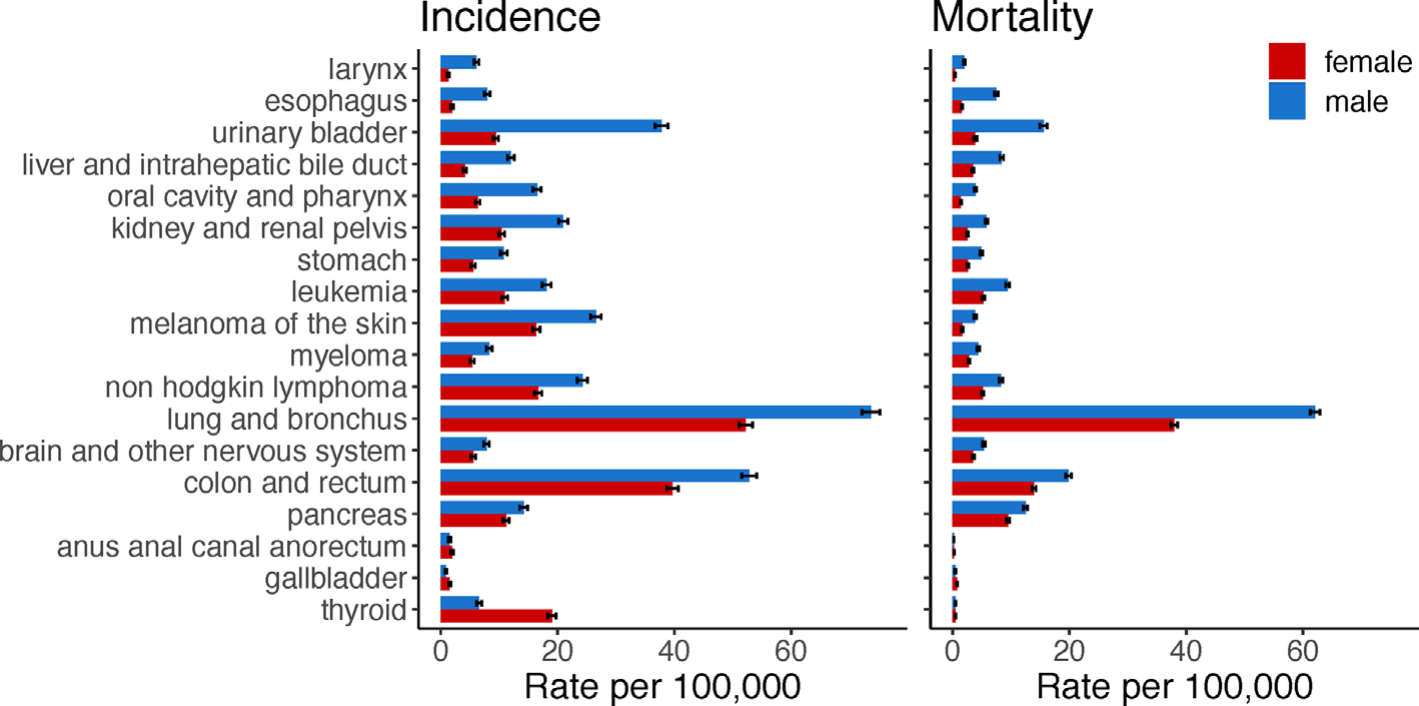
Cancers with sex disparity in incidence and mortality rates. Age-adjusted incidence and mortality rates per 100,000 individuals in the US were retrieved from the Surveillance, Epidemiology, and End Results explorer. Bars show the average rate from 2000 to 2017 and 95% confidence interval. Cancer sites are ordered according to the male-to-female incidence rate ratio; starting from cancer sites with higher incidence rates in males compared to females.

Sex disparities occur in cancer incidence and mortality (**Figure 1**). The mortality rate of all cancer sites combined is 214 for males, and 149 for females per 100,000, age-adjusted 2000–2017 average according to the Surveillance, Epidemiology, and End Results (SEER) program explorer. These sex disparities are apparent across a range of non-reproductive cancers and vary by age and race. In general, males have a higher incidence and a higher mortality rate than females for most cancer sites, including bladder, kidney, colorectum, liver, esophagus, head and neck, brain, skin, and blood (1). For cancer sites with higher incidence in males, the age-adjusted male-to-female incidence rate ratios range from 1.036 to 9.751, with a median of 1.588. Higher cancer incidences in females are found for breast, thyroid, cranial nerves, and a few of digestive system sites including gallbladder, anus, anal canal, and anorectum. Despite females having higher incidence for these cancers, they have better survival compared to males (1).

There are significant sex differences in therapeutic response and toxicity for many cancer types. Various chemotherapy regimens show higher toxicity, higher response rates, and longer post-treatment survival in women, including lymphoma (2), sarcoma (3), glioblastoma (4), lung (5, 6), and colorectal (7) cancers. There is also a growing literature showing cancer immunotherapy efficacy varies by sex (8–13). Poorer response rates are usually reported in females compared to males. However, controversial findings have been reported as the sex-based differences for immunotherapy response seems to depend on the cohort and cancer type analyzed as well as the immune checkpoint inhibitor agents used and whether they are used in combination with chemotherapy. Despite these known sex differences in therapeutic response, most treatment strategies, as well as drug development and selection, do not account for sex differences.

Studying both sex and gender is important to understanding differences in clinical manifestation between males and females and the molecular mechanisms involved; such considerations are now mandated as part of all NIH-funded research studies (14). Sex refers to a biological concept defined by the sex chromosome complement (15). Gender refers to a multidimensional social construct that may vary across societies and over time (16). Gender includes gender identity (the individual's self-perception and presentation), gender norms or roles (the individual's behaviors influenced by social and culture expectations) (17). The complex interplay between sex and gender is dynamic along an individual's life, influences not only cancer susceptibility and progression, but also how the individual perceives cancer, relates with health care, and adheres to treatments. Thus, it is essential to consider sex and gender in every stage of research and clinical care to improve prevention, diagnosis, and treatment for all individuals, being inclusive of gender minorities (18–20).

Sex and gender differences may influence cancer in different ways. Preventive behavior and exposure to risk factors were largely attributed to higher cancer incidence in males. However, gender-related occupational exposures (including heavy metals, and pesticides) and behavioral risk factors (including diet, tobacco, and alcohol consumption) can only partially explain higher cancer risk in males (21–23). Sex hormones play an important role in tumorigenesis and cancer susceptibility through several mechanisms likely to affect cancer stem cell self-renewal, the tumor microenvironment, the immune system, and the metabolism (24). In general, androgens have been associated with the higher cancer risk and mortality in males while estrogens observed to be protective in females (25–28). However, while sex differences in cancer may be modified by sex hormones, it is not completely explained by hormonal differences, and for most cancers over childhood and adolescent periods males also show higher incidence rates (29). Differential regulation of immune responses between males and females can also contribute to cancer susceptibility and outcomes. In general, females mount stronger immune responses than males, and sex differences in innate and adaptive immune responses occur throughout life (30). Differences in immune response and infection burden may also contribute to sex disparities of viral-related cancers. Human papillomavirus (HPV) infection rate differs by anatomic site and sex, and likely contribute to disparities in incidence of HPV-related cancers, including oral and anal (31). Other factors that might also contribute to sex differences in cancer include anatomy, physiology, body composition (lean and fat body mass), pharmacokinetics, and pharmacodynamics, which may affect drug metabolism, drug response, and drug toxicity (32, 33).

**Figure 2:**
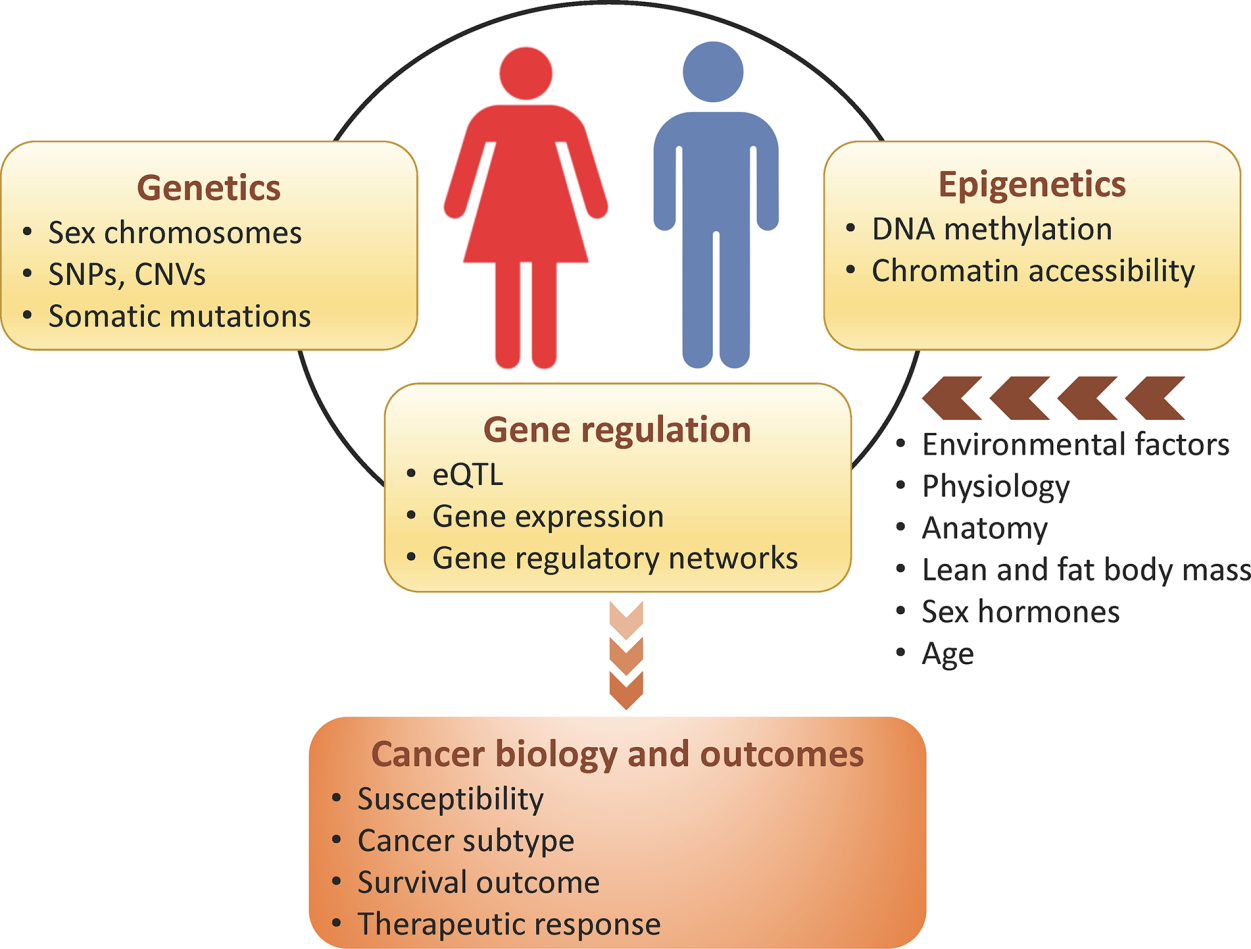
Sex differences in genomics, which can be modulated by host and environmental factors, influence cancer biology and outcomes.

Sex differences occur genome-wide, and are not restricted to sex chromosomes, can be independent of sex hormones regulation, and ultimately impact cancer biology and outcomes. More than half of the genes targeted by FDA-approved cancer drugs show sex-biased molecular signatures, including sex differences in somatic mutation, copy number, methylation, gene expression, and protein abundance (34) (**Table 1**). In this review, we discuss how genetics, epigenetics, gene regulation and expression may contribute to sex differences in cancer (**Figure 2**). We also describe how sex can be incorporated in genomics research and investigated through model systems. We finish with an example framework in colorectal cancer showing the impact of sex- and gender-based research to help us understand sex disparities in cancer and allow us to improve cancer prevention and treatment for both sexes.

**Table 1:** Examples of cancer drugs and their related-actionable genes, harboring sex-biased genomic alteration.

Gene	Molecular alteration	Sex bias	Cancer	Drug	Therapy type
*TOP2B*	methylation	female	BLCA	Valrubicin, Doxorubicin HCI liposome, Epirubicin	Chemotherapy (anthracyclines)
	mRNA	female	KIRP		
*PDCD1*	methylation	female	BLCA	Pembrolizumab, Nivolumab	Immunotherapy
	CNA	male	KIRC		
*AR*	protein	male	KIRC	Flutamide, Enzalutamide	Hormone therapy
					
*CTNNB1*	mutation	male	LIHC	Idelalisib	PI3K inhibitor
				Erlotinib	EGFR inhibitor
*EGFR*	mRNA	female	LUAD	Cetuximab, Erlotinib, Gefitinib, and Lapatinib	EGFR inhibitor
	methylation	female	BLCA		
*NF1*	mRNA	male	LUSC	Trametinib	MEK inhibitor
	mRNA	female	KIRP	Vemurafenib	RAF inhibitor
				Idelalisib	PI3K inhibitor
*CDKN2A*	mRNA	male	HNSC	Palbociclib	CDK inhibitor
	CNA	male	KIRC		
*TSC2*	methylation	female	KIRP	Everolimus, Temsirolimus	mTOR inhibitors
	methylation	female	KIRC		
*BRCA1*	methylation	female	HNSC	Olaparib	PARP inhibitor
	mRNA	female	KIRP		

Information obtained from Yuan et al. (34). CNA, copy number alteration; BLCA, bladder urothelial carcinoma; HNSC, head and neck squamous cell carcinoma; KIRC, kidney renal clear cell carcinoma; KIRP, kidney renal papillary cell carcinoma; LIHC, liver hepatocellular carcinoma; LUAD, lung adenocarcinoma; LUSC, lung squamous cell carcinoma.

### Sex Differences in Genetics

#### Sex Chromosomes

Genetic sex differences exist in the inherent inequality of the sex chromosomes, resulting in differences in the numbers of X chromosome genes between males and females and the presence of Y chromosome genes exclusively in males (35). A large part of the Y chromosome was lost during evolution, and only a small segment, the pseudoautosomal region (PAR), can recombine with the X chromosome. The Y chromosome evolved to retain genes with male-specific functions, as well as genes widely expressed involved in regulation of transcription, translation, and protein stability (36). Age-related loss of the Y chromosome in peripheral blood cells is a common somatic event in males and may be associated with higher risk of cancer, including leukemia, breast, and head and neck (37–40).

To reduce transcriptional dosage imbalance of X chromosome genes between the sexes, one of the X chromosomes in females is inactivated early in embryonic development and generally remains so throughout an individual's lifetime. Abnormalities in this process and reactivation of X-linked genes can occur in some tumors (41–43). The X inactive specific transcript (*XIST*) is a long non-coding RNA and major player during X chromosome inactivation, and loss of *Xist* in mouse hematopoietic cells results in X reactivation and genome-wide changes leading to leukemia (44).

X chromosome inactivation is incomplete, and some genes are expressed from both alleles resulting in important sex bias. Genes that escape X inactivation can have significant sex differences related to mRNA expression levels (45), patterns of transcription factor targeting (46), and frequency of somatic mutations (47). Across 21 cancer types, a higher mutation rate in males was identified for six "Escape from X-Inactivation Tumor Suppressor" (EXITS) genes: *ATRX*, *CNKSR2*, *DDX3X*, *KDM5C*, *KDM6A*, and *MAGEC3* (47). This finding showed that females might be protected for many cancers due to the biallelic expression of the EXITS genes and the need of a second hit to inactivate those. Functional experiments further support the evidence that EXITS genes can contribute to higher risk of many cancers in males, specifically for the X-linked lysine demethylase 6A (*KDM6A*, also known as *UTX*). *KDM6A* loss accelerates tumor initiation and progression of various *in vitro* and *in vivo* models for leukemia (48), lymphoma (49), pancreatic (50), and bladder cancer (51). Likewise, cancer risk differs between individuals with or without karyotype anomalies. Compared to the general population without karyotype anomalies, 1.34 increase in the risk of solid tumors was found in women with Turner syndrome, which is characterized by X chromosome monosomy (52). In men with Kleinfelter syndrome, characterized by the presence of two or more X chromosomes, the risk of solid tumors was reduced, but the risk of hematological tumors was increased, standardized incidence ratios of 0.66 and 2.72, respectively. This finding further supports the important role X chromosome plays in cancer etiology.

#### Genetic Variants

The association of genetic variants and sex effects is an understudied topic and challenging to reach power for sex-stratified or interaction analyses (53). However, evaluating genetic variants based on sex can reveal important sex disparities. Sex-biased effects of variants related to cell cycle and apoptosis have been described for many cancer types (54). Germline mutations in *TP53* are associated with increased cancer risk and earlier age at first-cancer diagnosis for females compared to males; female carriers have a 2.5- to 7-fold higher odds of having cancer than male carriers (55, 56). A single nucleotide polymorphism (SNP) found in the promoter region of *MDM2*, a negative regulator of the p53, is associated with increased risk of various cancer types in females but not in males, which was shown *in vivo* to be modulated by the estrogen-signaling pathway (57–59). Many genetic variants in immune-related genes can confer cancer risk or protection exclusively in one sex, including different types of variants such as SNP (60, 61), short tandem repeat (STR) (62), and copy number variant (CNV) (63).

There are important sex differences in pharmacokinetics and pharmacodynamics affecting drug metabolism and exposure, drug sensitivity and toxicity (32, 33). In general, clearance of various anticancer drugs is reduced by approximately 20% in females compared to males (33). There are also many examples of genetic variants in genes related to drug metabolism and detoxification that are associated with cancer risk. In acute myeloblastic and lymphoblastic leukemia, the deletion of *GSTT1* and a SNP in the *NQO1* gene confers higher risk to males than females (64). A SNP in the detoxifying-enzyme *SULT1A1* reduces the risk of bladder cancer in females but not in males (65). In lung cancer, genetic variants on *MTHFR* are associated with both increased or decreased risk in females, but not in males (66). Evidence also suggests interactions of *MTHFR* polymorphisms with both diet (vitamin B6, B12, and methionine) and tobacco smoking. In pancreatic cancer, interactions of SNPs in the drug metabolism genes *CYP1A2* and *NAT1* with tobacco and diet modify the risk of cancer differently in males and females (67). Taken together, these examples show that genetic variants might influence the sex divergence in cancer, and future studies need to consider the role of sex and gender in modifying both penetrance and expressivity of genetic variants.

#### Somatic Mutation

Sex-bias occur in mutation burden and in frequency of specific somatic mutations, which can include single nucleotide and copy number alteration (CNA). These sex differences in somatic mutation ultimately affect survival outcomes and therapeutic response.

Males have higher mutation burden (total number of mutations) for many cancer types, including lung adenocarcinoma (68), melanoma (69), urothelial cell, papillary renal cell, and hepatocellular carcinomas; difference in means by sex range from 0.16 to 2.2 (70). Among patients with high mutation count in melanoma (over 130 mutations), female patients have greater overall survival than males (69). In some cancers, mutation burden may be explained by sex differences in the efficiency of mismatch repair as suggested by the lower mRNA levels of DNA mismatch repair genes associated with high mutation burden in stomach and esophageal, kidney and liver cancers (70).

In a pan-cancer analysis using The Cancer Genome Atlas (TCGA) data, 15% of autosomal genes had sex-biased CNA, the majority of these were amplification and more prevalent in male tumors (70). Looking at each specific cancer type, eight harbored significant sex-biased frequency of CNAs: kidney clear cell, kidney papillary, head and neck, stomach and esophageal, liver, bladder, lung adenocarcinoma, and squamous cell cancers (34, 70). Sex-biased CNAs can be focal or cover long genomic segments, associated with differences in mRNA levels, and demonstrate differences in survival outcome. The authors identified 16 examples of sex-specific prognostic markers (70) (**Table 2**). For example, loss of *LATS1* was a marker of poor prognosis in females but not in males, and *UBAC1* loss was a marker of good prognosis in females but not in males.

**Table 2:** Examples of genomic alterations associated with survival outcome in a single sex or in both sexes with opposite effect.

Gene	Type of alteration	Prognostic value	Cancer	Reference
*RPL37A*	expression	only in females	colon	Li et al. (70)
*SRGAP1*	expression	only in males	colon	Li et al. (70)
*ACTL7B*	expression	both sexes, but in opposite directions	colon	Li et al. (70)
*TRRAP*	expression	both sexes, but in opposite directions	colon	Li et al. (70)
*LATS1*	CNA and expression	only in females	kidney clear cell	Li et al. (70)
*UBAC1*	CNA and expression	only in females	kidney clear cell	Li et al. (70)
*C16orf45*	CNA and expression	only in females	kidney papillary cell	Li et al. (70)
*LCMT1*	CNA and expression	only in females	kidney papillary cell	Li et al. (70)
*BRAF*	mutation	only in males	colorectal	Wangefjord et al. (71)
*BAP1*	mutation	only in females	kidney clear cell	Ricketts and Linehan (72)
*TP53*	mutation	only in females	colon	Warren et al. (73)
*miR-192*, *miR-206*, *miR-194*, and *miR-219*	expression	only in females	colorectal	Garufi et al. (74)

CNA, copy number alteration.

Specific cancer genes can also have sex-biased patterns of somatic mutation, including the lower frequency of *KRAS* mutation among males with colorectal cancer (75), higher mutation frequency of *PBRM1* and *KDM5C* in males and *BAP1* in females with renal carcinoma (72). Point mutations can also have sex-biased prognostic effect. *BRWD3* shows higher mutation frequency in HPV-negative head and neck cancer of females and associates with poorer overall survival (76). Overall, a genome-wide analysis of somatic mutation found that 0.8% of genes were prognostic in both sexes while 1.5% were prognostic in patients of only one sex (70).

Somatic mutations in therapeutic targets can be differentially present in males and females. β-catenin *(CTNNB1*) is more frequently mutated in men with hepatocellular carcinoma, and the activation of this proto-oncogene can affect sensitivity to EGFR, PI3K, AKT, and WNT inhibitors (34, 70). In contrast, the incidence of *EGFR* mutation is higher in females with non-small cell lung cancers (34, 77). The tumor suppressor gene *STK11* has higher mutation frequency in men with lung adenocarcinoma, whereas inactivating mutations in this gene may predict sensitivity to mTOR and SRC inhibitors (34). These examples highlight the need to carefully consider sex differences in cancer genomics during drug development and when defining treatment options.

**Figure 3:**
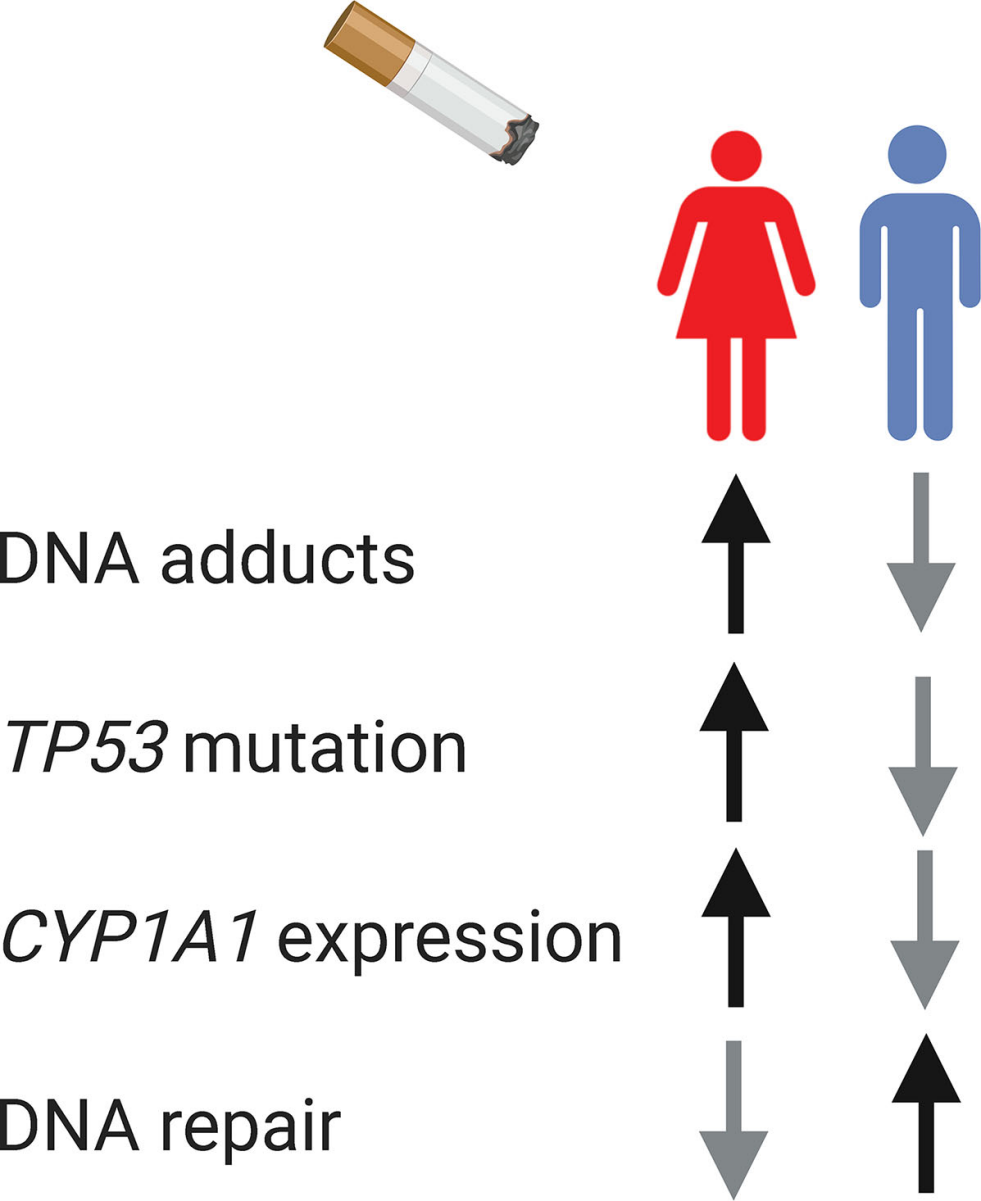
The interplay between sex, gender, and genomics. Biological sex and gender-influenced behavior, such as smoking, influence cancer genomics. In smoking-associated lung cancer, female lung tissue present higher level of DNA adducts, higher *TP53* mutation, higher expression level of the carcinogen-metabolizing enzyme CYP1A1, and less efficient DNA repair.

Gender-related behavior and exposures can also affect the gene mutation spectrum observed in males and females. In non-small cell lung cancer, both sex and smoking influence the mutation spectrum of *EGFR* (78) and *TP53* (79, 80). The frequency of G to T transversion mutation on *TP53* is higher among females (40%) than among males (25%–28%) (79, 80), which is a mutation signature of smoking-associated cancers (81). Tobacco may be more carcinogenic in females than males. Despite the lower levels of tobacco exposure, female lung tumor tissue present higher *TP53* mutation, higher level of DNA adducts, higher expression level of the carcinogen-metabolizing enzyme *CYP1A1*, and less efficient DNA repair (**Figure 3**) (79, 82–84). A combination of mechanisms can contribute to the sex differences in genetics, such as carcinogen exposures, DNA repair efficacy, and sex differential chromatin architecture (described in the *Sex Differences in Epigenetics* section).

## Sex Differences in Epigenetics

### DNA Methylation

Sex-biased methylation patterns have been observed across different human tissues, including blood (85–88), brain (89), liver (90, 91), rectal mucosa (92), muscle (93), and pancreas (94). Both the presence of the *SRY* gene and X chromosome dosage may influence sex differences in methylation level (95). Sex hormones may also influence methylation levels at specific gene regulatory regions, and in a tissue-specific manner, as observed in brain (96, 97) and liver (91). Furthermore, sex hormones can induce a stable methylation profile, establishing a sex-biased epigenetic memory maintained in the absence of the hormone.

In whole blood, 1,184 CpG sites in autosomes were found differentially methylated by sex, and further replicated in independent cohorts (86). Differences in male and female methylation patterns were enriched among imprinted genes and in CpG island shores (important for gene expression regulation), but not enriched in genes related with sex hormone biosynthesis, transport or receptors. By comparing sex-biased methylated genes in saliva and blood, an overlap of 81% was found for X chromosome genes and 8% for autosomes, confirming sex- and tissue-specific patterns of methylation (85). Genes differentially methylated in males and females were also associated with age, cigarette smoking, and alcohol consumption. As expected by the X-inactivation process, most CpG sites on the X chromosome had higher methylation levels in females compared to males. However, there are also higher methylation levels in males for some X chromosome sites. It is important to note that few studies have included the X chromosome in their analyses. A meta-analysis including 39 studies and several tissues, identified 184 autosomal CpG sites differentially methylated by sex (98). The sex-biased methylated CpG sites were located in the promotor of genes overrepresented in pathways important during cancer development, such as regulation of immune response, RNA splicing, and DNA repair. The magnitude of percent methylation differences between males and females found is usually small, with mean CpG site specific differences in methylation between males and females ranging from 3.7% to 17% (85, 98, 99).

Studies have identified sex-biased methylation patterns in cancer, including lung (100, 101) and colorectum (102). In leukemia, a genome-wide analysis identified 1,043 CpG sites differentially methylated by sex, of which 56 were located on autosomes and many known to play a role in tumor growth (103). A genome-wide analysis across 13 cancer types also reported sex-biased methylation patterns (34). On average, 236 genes with a sex-biased DNA methylation pattern were identified in the group of cancers defined as having strong sex-effect based on molecular signatures, and 10 genes in the weak sex-effect group of cancers. Most of the genes with sex-biased methylation also had sex-biased expression. The efficacy of methylation-based biomarkers for diagnosis and cancer risk prediction may be different for males and females with bladder cancer (104, 105). Moreover, radiation induces methylation changes in a sex- and tissue-specific manner (106, 107). Therefore, research focused on sex differences in DNA methylation is essential to improve treatment strategies and outcomes for all patients.

### Chromatin Accessibility

Sex differences in chromatin accessibility have been found in normal (108, 109) and tumor tissues (110). In whole blood, sex-biased chromatin accessibility was enriched for genes with sex-biased gene expression, and sex-biased regulatory genetic variants (108). Sugathan et al. developed genome-wide chromatin state maps for male and female mouse liver, and observed that the patterns of chromatin accessibility and histone marks differ by sex (109). The integration of these chromatin state maps with data for five transcription factors binding sites and gene expression showed that sex-biased gene regulation is mediated by a complex interplay between sex-biased chromatin marks in regulatory regions and differential transcription factor binding by sex. Sex-biased chromatin accessibility is partly established and maintained in response to sex-specific changes in patterns of plasma growth hormones (111).

In tumor tissues, sex-bias mutation frequency is observed for chromatin remodeling genes, such as *KDM6A* (47), *KDM5C*, *PBRM1*, and *BAP1* (72). Moreover, *BAP1* mutation is associated with changes in overall survival of clear cell renal cell carcinoma only for females (72). In analyzing the TCGA ATAC-Seq dataset across 23 cancer types, 2,534 peaks differed between males and females, of which 1,035 peaks were higher in males and 1,499 peaks were higher in females (110). Sex differences in chromatin accessibility were found in the promoter of genes located in both the X chromosome and autosomes, and in promoters of sex hormone receptors. Taken together, these studies show that both genetic and epigenetic patterns influence sex-biased gene regulation, and reinforce the need of integrative data approaches to study sex differences in gene regulation.

## Sex Differences in Gene Regulation

### eQTL

Sex differences in genetic regulation can contribute to sex differences in prevalence, progression, and severity of diseases (112). Ober et al. suggests that sex can be used as a variable that includes information on cellular, metabolic, physiological, anatomical, and behavioral differences between males and females, and can be modelled similar to a gene by environment interaction (112). Some loci have a regulatory role in one sex but not the other. For example, SNPs shared among sexes can be in regulatory regions that are sensitive to sex hormones. The impact of sex on gene regulation can be investigated through expression quantitative trait loci (eQTL) analysis. Sex-biased eQTL refers to an association between genotype and gene expression differing between males and females. Early studies have shown that sex-biased eQTLs are widespread affecting 12%–15% of eQTLs (113). An enrichment of sex-biased eQTL on the X chromosome compared to the autosomes has been found (108). Furthermore, sex-biased chromatin accessibility was enriched for sex-biased gene expression and regulatory variants. In analyzing 11,672 complex disease-associated SNPs as a function of sex and age in whole blood, 14 sex-biased eQTLs were identified (114). Most recent studies have reported few significant sex-biased eQTLs, and many not replicating across datasets (45, 115–117). These results may reflect the underlying biology with most genes unlikely to be influenced by sex or may indicate a combination of many small effect size and low power for genotype-sex statistical interaction tests (53).

A common and interesting finding is that most of the sex-biased eQTLs do not show sex-biased mean gene expression (45, 108, 113–115, 117). Sex-biased eQTLs may also be explained by sex differences in allelic direction or sex differences in gene expression variance. Most studies have investigated whole blood or lymphoblastoid cell lines (LCLs), and have not elucidated how sex-biased eQTL are associated with cancer. In hepatocellular carcinoma, 24% of the discovered eQTLs were sex-biased (118). This set included 24 genes under genetic regulation only in males and involved genes associated with Notch and PI3K/AKT signaling. Further studies in cancer will continue to elucidate the sex differential effect of genetic variants on tumor gene regulation.

### Gene Expression

Sex-biased gene expression is conserved for approximately 3,000 genes across mammals (119). Sex differential expression is found for both autosomal and sex chromosome genes across most human tissues (46, 117, 120–122), and cancer types (34). The largest expression differences are found for genes on the sex chromosomes, and the fold change of autosomal genes is generally small. The median fold change of autosomal sex-biased genes reported across 44 tissues was 1.04 (117). Despite X inactivation in females to compensate chromosome dosage, a number of X chromosome genes are differentially expressed by sex. Several X chromosome genes that escape inactivation are overexpressed in females in a tissue-specific manner (45). The opposite trend is observed for X chromosome genes that escape inactivation and are located in the pseudoautosomal region, showing higher expression in males compared to females. This pattern spans 29 normal human tissues, and it is also observed in sex-biased transcription factor targeting (46). Therefore, sex-bias mutation and expression of genes that escape from X-inactivation may contribute to higher susceptibility to certain cancers in males compared to females (47).

The number of differentially expressed genes varies by cancer type, extending up to 14% of the genes in clear cell renal cell carcinoma (34). Across cancer types, common pathways enriched for sex-biased genes include: immune response, metabolism, apoptosis and cell cycle, DNA repair and p53 (34). In hepatocellular carcinoma, sex differences are also found in several signaling pathways that are targets for anti-cancer therapies: PPAR is enriched for genes overexpressed in females, while PI3K, PI3K/AKT, EGFR, IL-2 are enriched for genes overexpressed in males (118). Other studies confirm that evaluating sex differential gene expression can help understand sex differences in cancer etiology (118, 123), distribution of molecular subtypes (124, 125), and outcomes (126–129). Sex differences in gene expression was also used to predict drug sensitivity in each sex (130).

New approaches to investigate sex effects in gene regulation and expression can provide better understanding of cancer in both sexes, which will be further exemplified by the significant sex differences in gene targeting by transcription factors discovered by gene regulatory network analysis (in the *Gene Networks* section). In glioblastoma, although a few number of genes are differentially expressed between males and females (34), the variance in tumor gene expression is sex biased (4). Using a join and individual variance explained (JIVE) analysis, Yang et al. identified five sex-specific molecular subtypes distinguished by gene expression and survival (4). The longest survival was associated with down-regulation of genes involved in cell cycle progression and in integrin signaling for males and females, respectively. This sex-specific pattern in expression was further associated with sex-biased chemotherapy sensitivity in a panel of male and female patient-derived glioblastoma cell lines. The authors also demonstrate the need to investigate sex-biased effect of somatic mutations to best define prognostic markers. Female patients with *IDH1* mutation, known as a good prognostic marker in glioblastoma, mostly clustered in a single group with longest survival, whereas male patients with *IDH1* mutation were distributed across all male clusters.

### miRNA

miRNAs are small non-coding RNAs involved in post-transcriptional regulation of gene expression and thereby regulate most cellular processes (131). MiRNA dysregulation is implicated in tumor development and progression. Similar to protein-coding genes, sex hormone receptors can bind and directly regulate miRNA transcription or indirectly through intermediate hormone-responsive transcription factors. In brain development, 162 miRNAs have sex-biased expression, 92 of which are estrogen-responsive, and relevant to differences in brain organizational structure by sex (132).

The chromosome location can also indicate sex divergence in miRNAs. A higher density of miRNAs is observed in the X chromosome compared to the Y and autosomes. The X chromosome contains 10% of the miRNAs, while the Y chromosome contains only two miRNAs (133). Several X-linked miRNAs are involved in immune regulation, and can also be involved in tumorigenesis (134). For example, miR-221 and miR-222 promote cancer cell proliferation (135). The miR-506-514 cluster is overexpressed in melanoma and involved in melanocyte transformation, melanoma growth, and sensitivity to BRAF inhibitors (136, 137). X-linked miRNAs directly target *PD-L1* or the transcription factors regulating *PD-L1* (138). High expression levels of miR-424(322) reverses chemoresistance by blocking the PD-L1 immune checkpoint (139). These miRNAs might escape X-inactivation, and therefore present higher expression levels in females. Further investigation may show the role of X-linked miRNAs in sex differences related to immunotherapy response.

Few studies have addressed the role of miRNAs in sex differences observed in cancer. Increased expression of miR-17 and let-7a was linked with familial female breast cancer compared to males (140). In metastatic colorectal cancer, the higher expression of miRNAs that regulate clock genes (miR-192, miR-206, miR-194, and miR-219) was associated with better overall survival in females (74). miR-137, involved in cell cycle control, is frequently methylated in squamous cell carcinoma of the head and neck, and higher methylation frequency in females was found compared to males (141). In performing genome-wide miRNA differential expression, Yuan et al. reported sex-biased expression of miRNAs and their potential role as regulators of sex-biased protein expression levels (34). However, the number of miRNAs differentially expressed by sex was small, and a median of seven miRNAs was found in the group of cancer types with strong-sex effect, such as thyroid, head and neck squamous cell, lung, papillary renal cell, and clear cell renal cell carcinomas. Future studies might still find miRNAs with sex differential targeting patterns despite limited differences in the miRNA expression levels between males and females.

### Gene Networks

**Figure 4:**
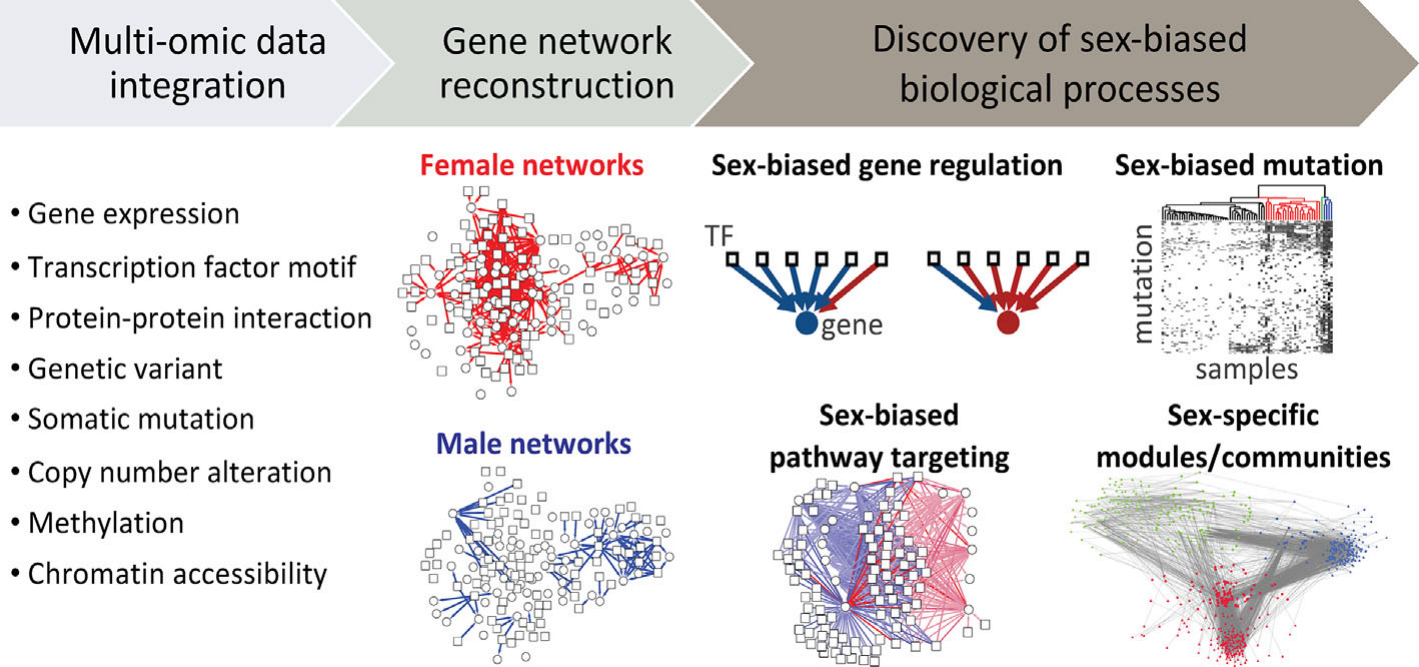
Using systems-based approach to study sex differences. Multi-omic data can be integrated to reconstruct gene networks that combine sex- and gender-biased effects and map how the connectivity and activity of genes differ between males and females. Analyzing the network topology and changes in network structure of males and females can provide insights into molecular mechanisms involved with sex differences in cancer. TF, transcription factor.

Although we have learned a great deal about sex differences through the study of individual genes, we have come to recognize that even the effects of single-gene mutations are moderated by networks of interacting regulatory and other elements within the cell. One distinct advantage of using network-based approaches is that they allow one to go beyond a list of altered genes, and map how the various cellular components interact with altered genes and with each other (142). Analyzing the network topology and changes in network structure of males and females can provide insights into the mechanisms associated with sex differences in cancer (**Figure 4**). The impact of a specific genetic or epigenetic alteration is rarely restricted to the activity of one gene product, and can propagate along complex gene networks. The term "sexome" describe the combination of sex-biased effects on gene networks, creating sex differences in connectivity and activity of genes (143). Decoding the sexome will help us better understand how sex interacts with gene regulation in cancer manifestation (144). Haupt et al. described sex-bias regulation of a p53 network across 12 non-reproductive cancer types (145). *TP53* mutation is not only more frequent in males compared to females, but X-linked negative regulators of p53 in wild-type *TP53* cancers including *UBE2A*, *MAGEA2*, and *UTP14A* show higher expression, and association with reduced survival of male patients. This suggests that having two X chromosomes might protect females from many cancers, a hypothesis further supported by the high number of non-expressed mutations among p53-associated X-linked genes, including *FLNA*, *MED12*, *HUWE1*, and *ATRX*.

Co-expression networks, in which genes are linked if their expression patterns are correlated (above some threshold), have been shown to differ in biologically meaning ways between the sexes. Studies have shown sex-based patterns of gene co-expression are recurrent across many non-reproductive tissues. Several sex-specific modules are identified when using Weighted Gene Co-Expression Network Analysis (WGCNA) to compare gene co-expression networks from males and females (146). Groups of genes with correlated expression are enriched for biological processes, including spermatid differentiation and development, epidermis and ectoderm development (120). Sex-specific modules of co-expressed genes may be modulated by gonadal hormones (147) and sex-biased eQTLs (148). Moreover, co-expression networks of autosomal genes may be altered by X-chromosome dosage (149).

Using gene regulatory networks to model the interactions between transcription factors and their target genes can identify distinct regulatory processes active in males and females, even when these differences cannot be found by analysis of individual gene expression. Using methods such as Passing Attributes between Networks for Data Assimilation (PANDA) and Linear Interpolation to Obtain Network Estimates for Single Samples (LIONESS) to model gene regulatory networks, sex differences in gene regulation was observed genome-wide and across all twenty-nine normal tissues analyzed (46). Several genes are differentially targeted by transcription factors between males and females, including those that are not differentially expressed. Transcription factor differential targeting can result from differences in methylation, chromatin accessibility, post-transcriptional modification of transcription factors, association of transcription factor complexes and co-factors. The sex hormone receptors were not the sole mediators of the sex-biased regulatory processes. The tissues with most sex-biased gene targeting are breast, thyroid, and brain, all of which have strong sex biases in cancer incidence and presentation. This resource of 8,279 regulatory networks across 29 normal tissues can be further investigated on whether there are regulatory processes in normal tissues that might explain sex differences in cancer incidence and development. Another study showed that gene regulatory networks can be used to identify sex-specific modules (or communities) (150). In breast tissue, female-specific modules include not only genes enriched for estrogen receptor signaling pathway, but also processes dysregulated in cancer such as regulation of cell-substrate adhesion, ERK 1/2 cascade, and response to type I interferon.

In analyzing gene regulatory networks in colon cancer, patterns of transcriptional regulation involving genes in the drug metabolism pathway distinguished colon cancer in males and females (151). These regulatory differences were found in primary colon cancer tissues before chemotherapy, and revealed that male and female patients are primed to respond differently to therapy. Importantly, drug metabolism genes were not differentially expressed by sex in the primary tumor, but higher transcription factor targeting of the drug metabolism pathway was associated with higher overall survival in females treated with chemotherapy; this pattern was not observed in males.

Taken together, these analyses of gene regulatory networks indicate that a combination of sex-biased factors may be reflected in the regulatory networks that control gene expression in each sex. This is consistent with substantial sex differences observed in methylation and chromatin accessibility. Males and females can regulate gene expression in different ways, and will likely respond differently to perturbations, such as environmental exposures, aging, somatic mutations, and therapeutic treatment. Even in the absence of strong sex differential expression, the study of regulatory networks can uncover latent sex differences in gene regulation, which might become important drivers of sex-biased manifestations during cancer development and progression.

## Incorporating Sex in Genomics Research

Many methods used in genomics research exclude sex chromosomes, and are not well-powered for sex-stratified or interaction analyses (53), largely because there are still many technical challenges to incorporate sex in genomics research. A framework to address sex differences in genomics analysis needs to (1) ensure adequate male and female ratio and sample size for powered analyses of each sex; (2) include the sex chromosomes in downstream analyses; (3) account for sequencing data mapping biases due to sequence similarity between the sex chromosomes; (4) statistically test for sex interaction effects. Sample size selection need to be carefully considered for powered analyses of each sex. For example, to study incidence in a disease with double incidence rate in males, the number of females enrolled need to be double the number of males in order to ensure similar statistical power for both sexes (152).

**Figure 5:**
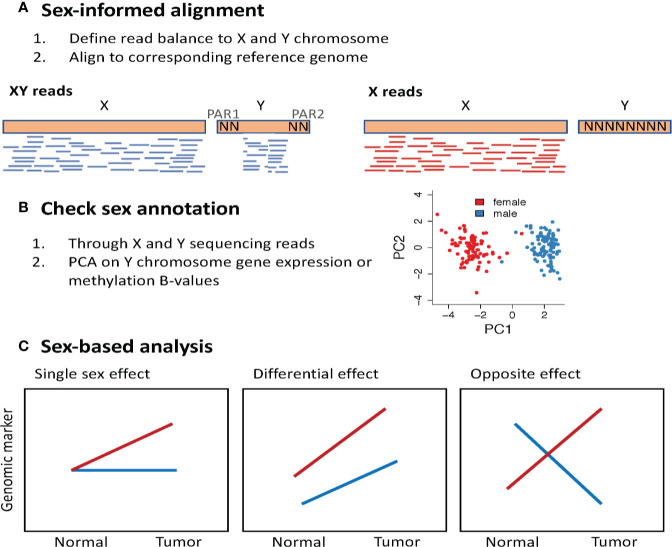
Methodological approach to study sex differences in genomics. **(A)** Perform sex-informed sequence alignment. The framework proposed in the XYalign tool (153), first identifies whether the sequencing reads are derived from an XX or XY genome, considering the balance of reads aligned to X and Y chromosomes. Next, sequencing reads from an XX genome are aligned against a reference genome with the Y chromosome masked, and sequencing reads from an XY genome are aligned against a reference genome masked for regions in the Y that are identical to X, the pseudoautosomal regions (PAR). **(B)** Use the genomic data to annotate biological sex based on sex chromosome complement. **(C)** Sex-based analysis may show how the effect of a genomic marker varies between males and females. PCA, Principal component analysis.

Sequence read mapping protocols do not account for the imbalance of sex chromosomes and the high sequence similarity between regions of the X and the Y chromosomes. This results in sequencing data that contains poor quality mapping of similar regions between the sex chromosomes, and spurious reads mapped to the Y chromosome in samples from XX genomes. A recent study showed that accounting for these artifacts in sequence mapping protocols can improve variant calling (153) and detection of sex differential gene expression (154). The XYalign is a sex-informed sequence alignment method that first identifies whether the sequencing reads derives from an XX or XY genome based on read balance, and then align the sequencing reads to a sex-appropriate reference genome (153). Sequencing reads from an XX genome are aligned against a reference genome with the Y chromosome masked, and sequencing reads from an XY genome are aligned against a reference genome, masked for regions in the Y that are identical to X (PAR1 and PAR2). It is worth noting that this is a new framework and has not yet been applied to large sequencing data efforts, such as the complete TCGA dataset. Another important quality control check is using the genomic data to annotate biological sex based on sex chromosome complement. This can be done using DNA sequencing data to define the balance of sequencing reads aligned to the X and Y chromosomes. Using RNA-Seq and methylation data, one can perform a principal component analysis using only the expression of Y chromosome genes (151, 155) or the methylation of X chromosome genes (98). A clear separation between male and female samples is expected when visualizing the first two principal components (**Figure 5**).

Statistical methods to detect differences between the sexes and interaction by sex have been reviewed elsewhere (152, 156). Sex-stratified analysis can be insufficient to appropriately estimate sex differences and detect sex interactions. Statistical interaction effects can be modeled to obtain the joint effect of two or more exposure variables on a disease outcome, such as the detection of interactions between sex and genetic variants to cause cancer.

## Investigating Sex Differences in Model Systems

Pre-clinical models are important tools to investigate how sex differences in genomics influence cancer etiology and manifestation. Several mouse models have been long used in cancer research, including models of spontaneous cancer or cancers induced by carcinogenic compounds, immunocompetent and immunodeficient mice transplanted with patient-derived xenografts. Additional models include transgenic mouse in which oncogenes and tumor suppressor genes are constitutively or conditionally manipulated using conventional approaches or the clustered regularly interspaced short palindromic repeats (CRISPR)-based genome editing (157). These mouse models can be used to evaluate sex-biased effects of genomic variation (4, 158–160). For example, Bahassi et al. evaluated the risk of spontaneous cancer or cancers induced by the carcinogenic compound 7,12-dimethylbenz[a]anthracene using mouse models with a genetic variant on *Chk2*, a cell cycle checkpoint kinase activated in response to DNA damage and involved in cell cycle arrest and DNA damage repair (54). In wild type mice, the percentage of males that develop spontaneous tumors is higher than females. However, in mice harboring a variant on *Chk2*, females develop spontaneous tumors with shorter latency and higher frequency.

Mouse models that uncouple the effects from gonadal secretions and sex chromosomes have been developed and can help us better understand the mechanisms associated with sex differences (161, 162). The four core genotypes mouse model is a good example on how to achieve these goals (163). In this model, the sex-determining region Y (*SRY*) gene is transferred from the Y chromosome to an autosome, and four animals are produced: XX and XY mice with ovaries, as well as XX and XY mice with testes. In this model, the gonad of the animal (testes or ovaries) and the genetic sex (XX or XY) are not related and allows to disassociate phenotype influenced from the sex hormones and sex chromosomes.

Using the four core genotypes mouse model, Kaneko and Li showed that the sex chromosomes are an independent factor associated with higher risk of bladder cancer in males, and the sex hormones amplify the sex-bias effects observed (51). Loss of the EXITS gene *Kdm6a* increases bladder cancer risk in female mice by reducing the expression of *Cdkn1a* and *Perp*, which are targets of p53 that induce cell cycle arrest and apoptosis. This was consistent with the clinical observation that mutations or reduced expression of *KDM6A* is associated with worse disease-free survival in female patients with bladder cancer but not in male patients.

While cancer cell lines are an indispensable research model, caution is needed when using cell lines to study sex effects. Several male-derived cancer cell lines have lost their Y chromosome, including many commercial cell lines, and therefore have eliminated the effect of sex chromosome complement on the investigational model (164). Using primary cancer cells can be a better alternative. For example, sex-biased chemotherapy sensitivity were observed in a panel of male and female patient-derived glioblastoma cell lines (4). Patient-derived cancer organoids can also be a potential model for studying sex differences. Three-dimensional organoids derived from individual patient's tumor have a high success rate and better recapitulate tumor complexity than two-dimensional cultured cancer cells, showing genomic, transcriptomic, and epigenomic concordance between organoids and their corresponding patient tumors (165–167). Moreover, organoid models have been used effectively for drug testing (168–170).

## Colorectal Cancer and The Impact of Conducting Sex-Based Research

**Figure 6:**
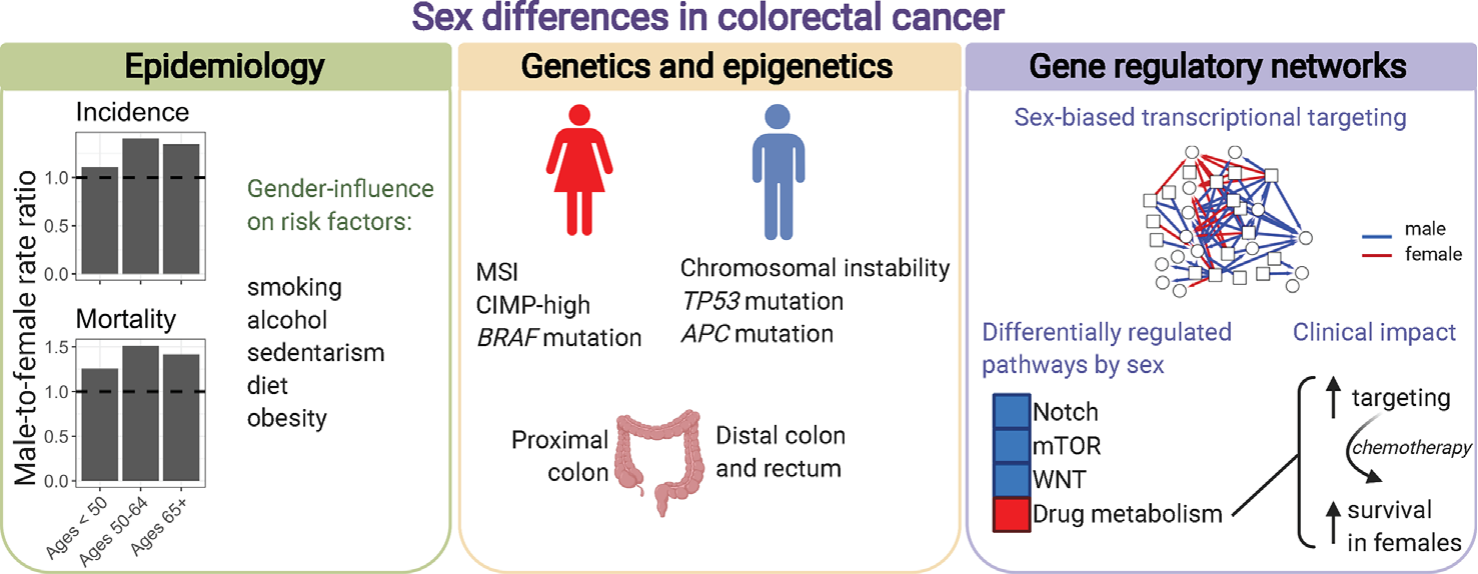
Sex differences in colorectal cancer. Males and females present differences in colorectal cancer incidence, mortality, anatomic site location, genetics, epigenetics, and transcriptional regulatory processes characterized by gene regulatory networks. Incidence and mortality rates per 100,000 individuals in the US were retrieved from the Surveillance, Epidemiology, and End Results explorer (2000–2017).

Here, we will focus on colorectal cancer as an example to emphasize the need to increase awareness of how sex influences cancer, and the importance of conducting sex-based research to better achieve the goals of precision medicine (**Figure 6**). Sex differences exist in the incidence, clinical and pathological presentations of colorectal cancer. However, these sex differences are not considered during prevention and treatment.

Colorectal cancer is the third most common cancer, and the third leading cause of cancer death among males and females (171). Overall, males with colorectal cancer have worse survival than females (age-adjusted male-to-female hazard ratio of 1.124) (1). Across races and geographical regions, males have higher incidence of colorectal cancer than females (172). In the United States, the age-adjusted male-to-female incidence rate ratio (IRR) is 1.287 (1). Incidence rates vary according to anatomic subsite and age, with significant changes observed for distal colon cancers (1, 173). Specifically, for sigmoid colon male-to-female IRR increases from 0.869 at 15-39 years, to 1.335 at 40-64 years, and to 1.526 after 65 years (1). Compared to distal colon, patients with proximal colon cancer are predominantly females (174). Biological sex differences, and gender-related behavior can contribute to observed differences in incidence by sex. Known risk factors for colorectal cancer include smoking, alcohol consumption, sedentarism, diet high in red and processed meat, and obesity (175). For example, overall obesity, as measured by high body mass index, is a known risk factor for colorectal cancer with larger associations observed in males compared to females. Studies have shown that not only obesity but the pattern of weight gain throughout life influences the cancer risk. Early life obesity is the primary risk factor for colorectal cancer in females, and adult weight gain in males (176). Sex hormones can also influence colorectal cancer incidence. In general, estrogens are shown to be a protective factor for colorectal cancer in females, whereas testosterone is associated with increased risk in males (25, 28, 177, 178). A high estrogen/testosterone ratio is associated with lower relative risk of colorectal cancer in postmenopausal women, but higher risk in men (179).

Sex differences are observed for many molecular features that are associated with the presentation, progression, and treatment response of colorectal cancer. In general, female sex is associated with microsatellite instability (MSI), CpG island methylator phenotype (CIMP)-high, and *BRAF* mutation (180). Male sex is associated with chromosomal instability, and mutation on *TP53*, and *APC.* MSI, observed in approximately 15% of colorectal cancers, is characterized by a hypermutable phenotype due to a deficient DNA mismatch repair system (181). This molecular phenotype, more frequently observed in females, is a marker of better prognosis and resistance to chemotherapy (182, 183). MSI in sporadic cancer is generally caused by hypermethylation of *MLH1*. MSI is also the main molecular feature to identify the most frequent form of hereditary colorectal cancer, Lynch syndrome, caused by germline mutation in one or more DNA mismatch repair genes. Germline mutation in these genes is associated with greater lifetime colorectal cancer risk in males compared to females (74% versus 30%) (184). Another example of sex-biased genetic feature is observed for *KRAS*, a major driver of colorectal tumorigenesis, and marker of resistance to EGFR-targeted therapy (185). While 30%–40% of colorectal cancers carry a *KRAS* mutation, a higher mutation frequency is found in females compared to males, with pronounced differences for tumors in the proximal colon (75, 186, 187). Moreover, carcinogens may act in a sex-biased manner, supported by an enrichment of a specific type of mutation (G to C transversion) in rectal tumors from females (187).

When comparing large-scale genomic features between males and females with colorectal cancer, including mutation, CNA, methylation, protein, miRNA and gene expression, only a small number of features show significantly sex differences (34, 70). However, similar genomic backgrounds can have different effects in males and females. There are many examples of prognostic and predictive biomarkers that are sex-specific or of greater value to one sex (71, 73, 160, 188). Therefore, the combination of environmental exposures and small size-effect genomic factors can work together on modifying the risk and disease presentation in each sex, and consequently be reflected in the organization of gene networks. In a study modeling gene regulatory networks for each of 1,308 patients with colon cancer, although gene expression only differed significantly in sex chromosome genes, gene regulatory networks exhibited marked sex differences in transcriptional regulatory processes (151). Genes more strongly targeted by transcription factors in males were enriched for pathways with key roles in colon cancer development, including the Notch, mTOR, and WNT signaling pathways. Moreover, genes in the drug metabolism pathway presented differential transcriptional regulation by sex. These sex-linked regulatory patterns were found in primary colon cancer tissues before chemotherapy, and were associated with higher overall survival in females treated with chemotherapy, but not in males. The genes with the largest regulatory sex differences belong to the glutathione S-transferase (GST) family involved in removing xenobiotics. Indeed, females with colorectal cancer have better survival benefit from adjuvant chemotherapy (7), but also higher toxicity (189, 190). Sex differences in drug metabolism and elimination, not only in renal and metabolic clearance (33) but also in gene transcriptional targeting in the tumor (151), suggest adjustment of drug doses by sex. Clinical trials are not designed to identify different optimal doses for both males and females, but dose modification by sex should be further evaluated for targeted therapies and some checkpoint inhibitors that are administered at fixed doses, and also for chemotherapy and antibodies that are dosed according to body surface area and weight.

## Discussion

There is an extensive body of clinical and epidemiological evidence for variability in cancer associated with sex and gender and a growing body of multi-omic data that demonstrate the presence of genome-wide sex differences in cancer beyond those affecting reproductive tissues. Males and females respond differently to environmental exposures and genetic and epigenetic alterations. Thus, genomic features associated with cancer etiology, prognosis, and therapeutic response may have differential effects in males and females. For example, changes in gene expression or mutation can be prognostic and predictive markers in only one sex or in opposite directions between the sexes (**Table 2**). Differences between male and female cancers are observed when analyzing several omic data types, including mutation, copy number, DNA methylation, chromatin accessibility, and expression of mRNA, miRNA, and protein. Integrating multi-omic information can better inform molecular mechanisms involved with sex differences in cancer. For example, analysis of sex-specific gene regulatory networks can identify biological processes differentially regulated by sex, and how sex-biased patterns associate with sex differences observed during tumorigenesis and clinical outcomes.

Despite the overwhelming evidence that sex influences cancer incidence, progression, and therapeutic response, the widely used paradigm in precision medicine generally ignores the sex of the individual. Further, there remain conceptual and methodological gaps to incorporating sex in research and in clinical practice (191, 192). Although the growing literature on sex differences in cancer manifestation and in cancer genomics provide strong arguments for implementation of sex-aware precision therapy, additional data are needed to fill the gaps in our understanding. This will require that sex be explicitly considered as a key variable in the design and conduct of both preclinical and translational research. Continuing to investigate sex and gender differences in cancer will inform sex-specific strategies for cancer prevention and early diagnosis and will lead to more refined precision medicine therapeutic strategies that will improve treatment and outcomes including survival.

## Author Contributions

CL-R wrote the first draft of the manuscript. All authors contributed to the article and approved the submitted version.

## Funding

CL-R, and JQ are supported by a grant from the National Cancer Institute, National Institutes of Health, R35 CA220523. JQ is also supported by U24 CA231846. DLD is supported by grants from the National Heart, Lung, and Blood Institute (5P01HL105339, 5R01HL111759, and 5P01HL114501) and by a BWH First-In-Women Precision Medicine IGNITE award.

## Conflict of Interest

The authors declare that the research was conducted in the absence of any commercial or financial relationships that could be construed as a potential conflict of interest.
